# Supplementary material for: Presence of Infection by Mycobacterium avium subsp. paratuberculosis in the Blood of Patients with Crohn’s Disease and Control Subjects Shown by Multiple Laboratory Culture and Antibody Methods
Source: Microorganisms. 2020 Dec 21;8(12):2054. doi: 10.3390/microorganisms8122054 (PMC7767509; doi:10.3390/microorganisms8122054)
Supplement: Supplementary file 1 [file microorganisms-08-02054-s001.pdf]

Supplementary Figures

Supplementary Figure 1. Representative plates showing plaque numbers observed in phage assay positive PBL samples tested during June 2018.

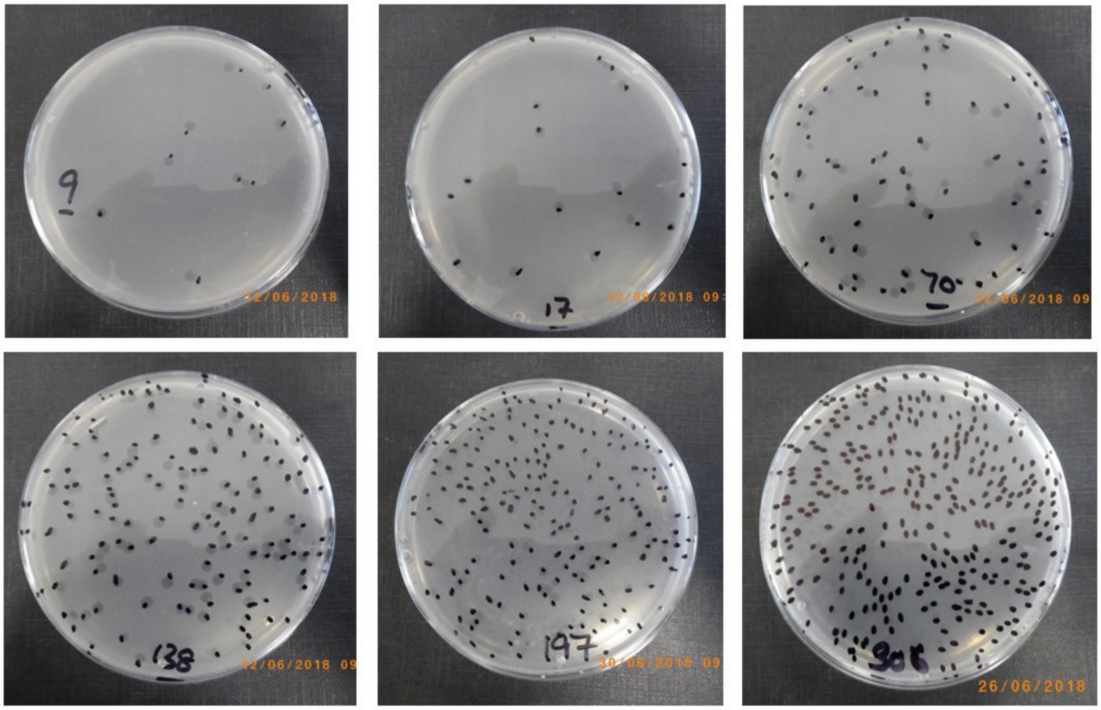

Overall, plaque numbers ranged from 1-500/half PBL sample (other half of sample was cultured in Pozzato broth).

Supplementary Figure 2. Mean plaque numbers ± SEM obtained for phage assay positive PBLs from CD patients, UC patients, Non-CD or UC with autoimmune disease, and non-CD, non-UC and non-autoimmune disease controls.

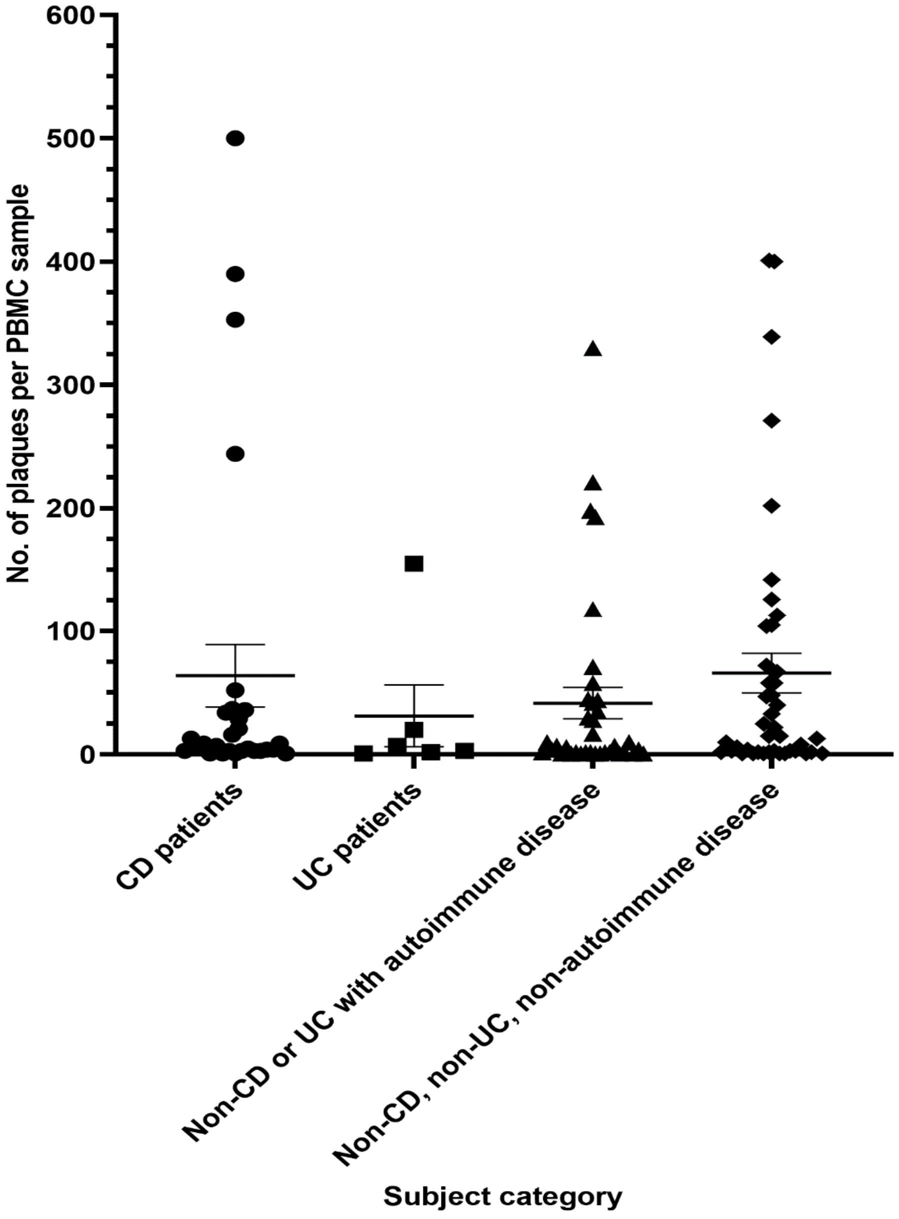

ANOVA indicated no significant difference between mean plaque numbers of the four subject categories (CD patients 63.85 plaques, UC patients 31.33 plaques, Non-CD or UC with autoimmune disease 41.67 plaques, and Non-CD, non-UC and non-autoimmune disease controls 66.05 plaques, p-value = 0.6604). Variance within the Non-CD or UC with autoimmune disease group was significantly less than the CD patient subject category only (p-value = 0.0021).

Supplementary Figure 3. Impact of length of PBL sample transit time between blood draw in USA and commencement of testing at Queen's University Belfast, UK, on plaque numbers obtained with the phage assay.

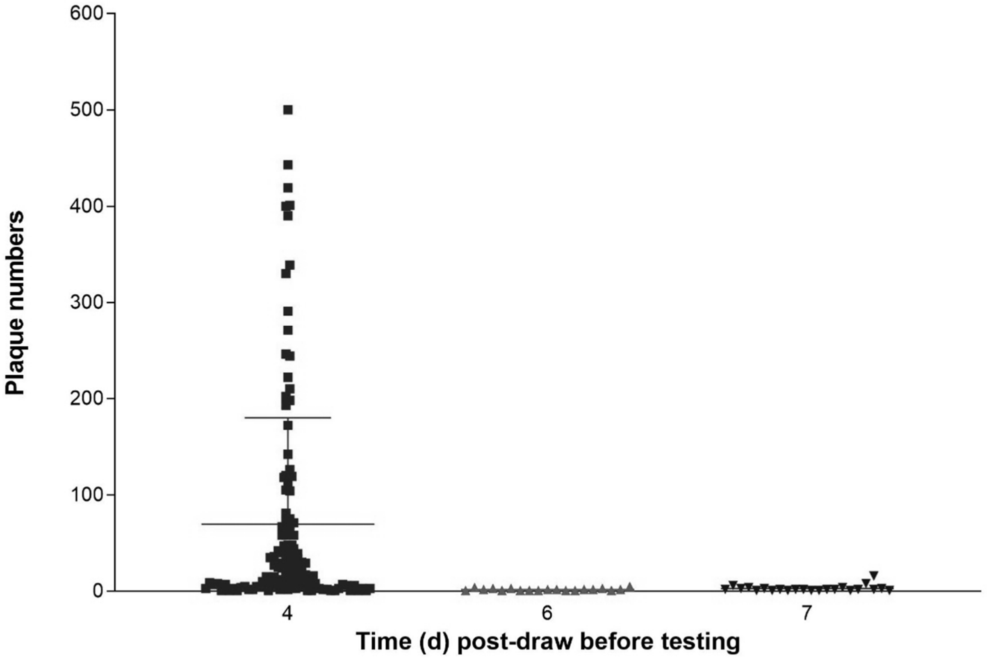

One-way ANOVA indicates significantly higher plaque numbers (p-value = 0.0012) for phage assay positive PBL samples tested at 4 days post-draw compared to phage assay positive PBL samples tested at 6 or 7 days post-draw.
